# Strengthening vaccines and medicines manufacturing capabilities in Africa: challenges and perspectives

**DOI:** 10.15252/emmm.202216287

**Published:** 2022-06-27

**Authors:** AbdulRahman A Saied, Asmaa A Metwally, Manish Dhawan, Om Prakash Choudhary, Hani Aiash

**Affiliations:** ^1^ National Food Safety Authority (NFSA) Aswan Egypt; ^2^ Ministry of Tourism and Antiquities Aswan Egypt; ^3^ Department of Surgery, Anesthesiology, and Radiology, Faculty of Veterinary Medicine Aswan University Aswan Egypt; ^4^ Department of Microbiology Punjab Agricultural University Ludhiana Punjab India; ^5^ Trafford College, Altrincham Manchester UK; ^6^ Department of Veterinary Anatomy and Histology, College of Veterinary Sciences and Animal Husbandry Central Agricultural University (I), Selesih Aizawl Mizoram India; ^7^ Department of Family Medicine Suez Canal University Ismailia Egypt; ^8^ Department of Cardiovascular Perfusion SUNY Upstate Medical University Syracuse NY USA; ^9^ Department of Medicine SUNY Upstate Medical University Syracuse NY USA; ^10^ Department of Surgery SUNY Upstate Medical University Syracuse NY USA

**Keywords:** Africa, COVID‐19, manufacturing, mRNA vaccine, vaccines, Economics, Law & Politics, Microbiology, Virology & Host Pathogen Interaction

## Abstract

Africa carries a high burden of infectious diseases. Every year, millions of Africans contract tuberculosis, malaria, and many other diseases. Malaria kills hundreds of thousands of children under the age of five years annually. More than 11,000 people died during the 2014–2016 Ebola outbreak in West Africa; still, occasional cases of Ebola, as well as monkeypox, periodically appear in the Democratic Republic of Congo. Since most of the African countries gained their independence during the 1960s, the continent has relied heavily on the outside world for diagnostics, medicines, vaccines, personal protective equipment, and other medical supplies. Africa consumes nearly 25% of the globally produced vaccines but imports 99% and 95% of its vaccines and medicines, respectively. The 55 African countries were not able to ensure the health of 1.3 billion Africans during the COVID‐19 pandemic but had to rely on other global initiatives and other countries for help and support. However, the pandemic and the shortage of vaccines may have been the much‐needed trigger for this situation to change. “When misfortunes increase, they erase each other.” *Naguib Mahfouz (1911–2006)*.

## Graphical abstract

In this Commentary, A. Saied & colleagues discuss the current situation in Africa regarding vaccine manufacturing and distribution, as well as challenges and perspectives to achieve a greater independence.

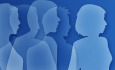

## Africa: a history of importing medical supplies

During 2017 and 2018, more than 120 disease outbreaks were reported on the African continent. More than 10 million Africans died unnecessarily as a result of not being able to get antiretroviral medications that are easily available in wealthier countries (Happi & Nkengasong, 2022). Since many African countries gained their independence, they have relied heavily on the outside world for diagnostics, medicines, vaccines, personal protective equipment, and other medical supplies (Happi & Nkengasong, 2022). This continuing reliance on other countries contributes to a lack of trust in African countries, from both within and outside. In particular, the lack of vaccine manufacturing capacity was one of the most significant obstacles during the COVID‐19 pandemic. However, merely increasing supplies of COVID‐19 vaccines to African countries is not a sustainable solution (The Lancet Infectious Diseases, 2022). The continent needs to build up its own capacity to produce and distribute drugs and vaccines.

**Figure 1:**
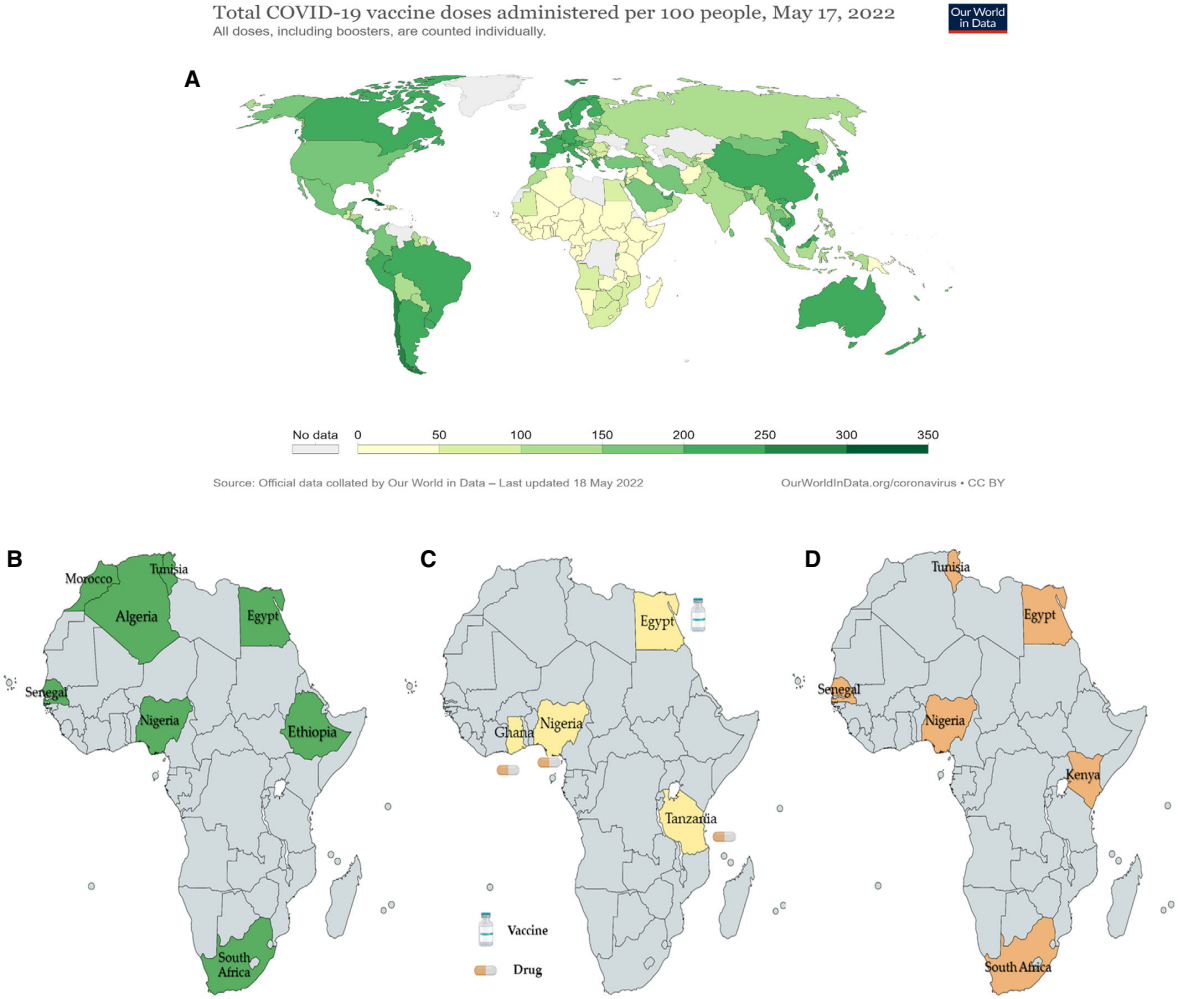
(A) Map shows the number of vaccine doses administrated per 100 people. It is interesting to note that Africa is struggling to vaccinate their populations, while other countries have already provided third booster doses to a large number of people. (B) Vaccine manufacturing capacity in Africa. (C) Tanzania (2018), Ghana (2020), Nigeria (2022), and Egypt (2022) regulatory systems have reached ML3. (D) The six African countries that have joined to mRNA technology transfer hub.

Africa is the second most populous continent after Asia, with 1.3 billion people. It has a vaccination rate of only 16% against COVID‐19 (Fig 1A), which is far lower than on every other continent (60–80%) (Davies, 2022). Although the COVID‐19 Vaccines Global Access (COVAX) has played a critical role in achieving this vaccination rate, many scientists, public health experts, and organizations have realized that reliance on a few companies or donations from other countries is both restrictive and risky during the pandemic. The low vaccination rate is largely attributed to the limited capacity of Africa's vaccine production (Editorial, 2022). The few African vaccine manufacturers are located in eight African countries: Egypt, Tunisia, Algeria, Morocco, South Africa, Senegal, Nigeria, and Ethiopia (Fig 1B; Table 1). They have varying capabilities but mostly “fill and finish” imported vaccines owing to a lack of local scientific capacity as well as unreliable supply chains (Ekström *et al*, 2021). The African Development Bank has been funding the Africa Centres for Disease Control and Prevention (Africa CDC) and pledged to spend US$3 billion over the next 10 years to enhance vaccine manufacturing and meet the African Union's target of producing 60% of the needed vaccines on the continent by 2040 (Editorial, 2022). With an additional increase in the local production of pharmaceuticals to 70% by 2030 according to a 2030 Pharmaceutical Action Plan/Continental Vision for Africa. Long‐term investment to create more vaccine production capacity and efficient oversight and regulatory mechanisms across Africa are required to ensure the long‐term success and supply of vaccines and drugs.

**Table 1:** Vaccine manufacturing capacity in Africa.

S.	Country	Vaccine manufacturing facilities	Established in	Manufacturing stage
1.	Egypt	VACSERA	1881	Fill & finish Pack & label
		Biogeneric Pharma	2005	Research
		Minapharm	1958	Research Fill & finish
2.	Tunisia	Institut Pasteur Tunis	1956	Drug substance manufacturing Fill & finish
3.	Algeria	Saidal	1982	Drug substance manufacturing Import for distribution
4.	Morocco	Sensyo Pharmatech	2024	Fill & finish
		Institut Pasteur Du Maroc	1929	Import for distribution
		Galenica	1978	Drug substance Fill & finish Pack & label
		Sothema	1976	Pharmaceutical manufacturing company
5.	Senegal	Institut Pasteur De Dakar	2009	Drug substance manufacturing Fill & finish Pack & label
6.	Nigeria	Innovative Biotech Ltd	2005	Research & development
		Biovaccines Nigeria Limited	2005	Research Pack & label
7.	Ethiopia	Ethiopian Public Health Institute	1995	Pack & label Import for distribution
8.	Ghana	Ghana Health Ministry	2024	Drug substance manufacturing
9.	Kenya	Afrigen	2022	Drug substance
10.	Uganda	Dei Biopharma	2022	Drug substance manufacturing
11.	Rwanda	Rwanda Biomedical Center	2023	Drug substance manufacturing
12.	Botswana	Botswana Baylor Children's Clinic	2026	Drug substance manufacturing
13.	South Africa	Aspen	1997	Fill & finish
		Biovac	2003	Research & development Drug substance manufacturing Fill & finish Pack & label Import for distribution

https://theconversation.com/making‐covid‐vaccines‐in‐africa‐advances‐and‐sustainability‐issues‐182683

## African regulatory bodies: a step forward

Medicinal product regulation is essential for healthcare systems to guarantee access to high‐quality vaccines, medicines, and other health products. On March 30, 2022, the World Health Organization (WHO) declared that the regulatory authorities for medical products in Egypt (Egyptian Drug Authority; EDA) and Nigeria (National Agency for Food and Drug Administration and Control; NAFDAC) reached Maturity level 3 (ML3) after formal evaluation (WHO, 2022a); they joined Ghana and Tanzania (Fig 1C) as countries with ML3 regulatory systems. Egypt has reached ML3 in the regulation of vaccines both locally produced and imported, and Nigeria has reached ML3 in the regulation of pharmaceuticals and imported vaccines.

The WHO's evaluation of regulatory authorities is based on the Global Benchmarking Tool, which compares regulatory functions with a set of more than 260 indicators to determine their maturity and functionality. ML3 is the second highest in the four‐tiered scheme, and it means that these authorities will be eligible for inclusion in the transitional WHO‐Listed Authorities (WLA) that meet WHO and other international standards for ensuring access to safe, effective, and high‐quality medical products. The model allows regulatory authorities, the WHO Prequalification Program, and procurement agencies to rely on recommendations of other authorized bodies for their decision‐making (WHO, 2022b). Notably, this enables more efficient use of limited resources as it makes it easier for other regulatory authorities on the continent to rely on the recommendations and decisions of ML3 agencies. Including Egypt and Nigeria in the WLA thus helps to produce and disseminate safe and effective medical products throughout the continent with global trust and, more importantly, will lead to more innovation and investments (Table 2).

**Table 2:** Consequences of entering African countries into ML3 and inclusion into WLA.

1.	Promotes good regulatory processes to ensure that essential medicines and vaccines are available in a safe, effective, high‐quality, and affordable manner.
2.	Improve the efficiency with which regulatory agencies operate to ensure timely access to high‐quality medications and vaccine prequalification.
3.	Provide effective and high‐quality regulatory oversight to support local medicinal product manufacturing that meets international standards.
4.	Facilitate other regulatory agencies' trust, collaboration, recognition, convergence, and harmonization.
5.	Boost pharmaceutical trade by increasing trust among national regulatory authorities, manufacturers, healthcare providers, and consumers.
6.	Promote quicker product authorization and safety monitoring after authorization (Pharmacovigilance; PV).
7.	Increase production capacity.
8.	Transparency, legitimacy, and public trust.
9.	WHO provides technical assistance and training to enable countries to implement global guidelines to meet their specific health needs.

## COVID‐19 sparked the birth of the African vaccine industry

Egypt has worked hard from the beginning of the COVID‐19 pandemic to become self‐sufficient in COVID‐19 vaccines and medicines (Saied, 2022). VACSERA, the Egyptian Holding Company for Biological Products and Vaccines signed agreements with Sinovac, a Chinese biopharmaceutical company, to produce the VACSERA‐Sinovac vaccine locally (Saied, 2022). Egypt is one of the few African countries with vaccine production capabilities and has extensive expertise with diseases such as polio, Rift Valley Fever and rabies. However, VACSERA has traditionally focused on late‐stage production, mostly “fill and finish” vaccines and drugs for distribution. In addition, Algeria, Morocco, Rwanda, Senegal, and South Africa have either signed agreements or memorandums of understanding for manufacturing COVID‐19 vaccines or have already started production. Côte d'Ivoire, Ghana, Kenya, and Nigeria have shown interest in vaccine production too. Within the next two decades, African Union member states aspire that 60% of Africa's routinely used vaccines will be manufactured on the continent (Editorial, 2022).

In response to enormous global inequities in COVID‐19 vaccine distribution, many initiatives were launched to provide low‐ and middle‐income countries (LMICs) with vaccines (Saied, 2022). The WHO has established an mRNA tech transfer hub in Africa—at Afrigen Biologics and Vaccines, a biotech company in Cape Town, South Africa—to help LMICs produce their own COVID‐19 vaccines to international standards. In addition, WHO, the Medicines Patent Pool, COVAX, the African Union, and the African CDC are among the partners in the global mRNA technology transfer hub. In February 2022, the hub chose Egypt, Nigeria, Kenya, Senegal, South Africa, and Tunisia as recipients of mRNA technology (Fig 1D). It is one of the strategies for preparing for future health challenges and ensuring that LMICs get access to medicines and vaccines. In the six selected African countries, the hub will share technology and technical knowledge for the development and licensing of mRNA vaccines with local companies. So far, however, Afrigen Biologics company has produced only microliters of Moderna's COVID‐19 mRNA vaccine (Saied, 2022) as a starting point for manufacturing mRNA vaccines in Africa with local resources.

## Africa and mRNA vaccine makers

Separate to WHO's efforts, Moderna, with assistance from the US Government, has signed a Memorandum of Understanding with Kenya to build a new mRNA vaccine production plant for US$500 million. This would be the first such factory in Africa, with close to 500 million doses of mRNA vaccines produced annually. Although there are few details available about this venture, Moderna staff will, at first, operate the plant to produce mRNA vaccines, including the COVID‐19 vaccine, starting with active ingredients and potentially expanding to fill‐finish.

BioNTech plans to send container‐based COVID vaccine factories to Rwanda and Senegal to reduce the building time by at least a year, with vaccines expected to become available in late 2023, just 12 months after the containers are installed. These mobile mRNA vaccine factories are specially designed shipping containers (BioNTainer)—each measuring 800 sq.m—capable of producing 50 million doses of the Pfizer/BioNTech COVID vaccine annually. The facilities will be supervised and operated by BioNTech staff first. Over time, ownership and expertise will be transferred to local companies to take over the complex vaccine‐making process after training local staff. Furthermore, BioNTech and Pfizer will provide the vaccine's active ingredient to Biovac in South Africa, which will fill‐finish, and distribute 100 million vaccine doses to the African Union each year starting this year. Ultimately, Afrigen and Biovac can manufacture as many as 500 million doses a year, although capacity will likely increase once other companies on the continent learn how to produce the vaccine.

## Challenges and perspectives

There are still considerable challenges to building vaccine production capacities in Africa, chiefly the lack of trained staff and public health infrastructure (Saied, 2022). Additionally, vaccine uptake could be hampered by a lack of adequate maternal‐health facilities, lack of access to both reliable information on vaccines and the vaccines themselves, and people's long‐held cultural beliefs.

Moreover, maintaining an unbroken cold chain from production to patient is a top imperative, as vaccines must be kept at low temperatures to keep them efficient. It will also be necessary to improve the cold chain infrastructure at medical facilities. By way of example, just 48% of health institutions in Ethiopia have a reliable cold chain. Inadequate coordination with local agencies, a lack of monitoring bodies, insufficient monitoring and recording of vaccine temperature, and a lack of financial support for the purchase of vaccines were identified as additional challenges. In addition, the administration of vaccinations outside the cold chain needs more detailed investigation and oversight (Fahrni *et al*, 2022).

During the 2014–2016 Ebola outbreak, the Merck (Ervebo) vaccine had to be kept at −70 °C during transport. As a solution, the Arktek Cold Storage Device was used in Sierra Leone to transport vaccines that saved the lives of hundreds of thousands of Africans. The Arktek Passive Vaccine Storage Device (PVSD) is based on decades‐old technology originally developed to safeguard spacecraft from high temperatures. The PVSD is a super‐insulated container that can keep vaccines safe for up to 1 month at 2°C to 8°C, using only ice packs. For the Ebola vaccine, the device was modified to handle −80°C (with some leeway in maintaining a storage temperature of −60°C or colder) with a payload of approximately 200 vials (Jusu *et al*, 2018). The modified Arktek devices proved invaluable for shipping and backup storage during power outages or freezer malfunctions. It is a great solution for dealing with poor infrastructure, lack of cold chain facilities, lack of specialized equipment required for storing vaccines at extremely low temperatures, power interruptions, and freezer equipment failure. Therefore, these devices could play a crucial role in facilitating the distribution of mRNA vaccines in Africa against SARS‐CoV‐2 and other viral pathogens.

Governments and nongovernmental organizations (NGOs) must provide long‐term support for research and development, focusing on diagnostics, treatments, and vaccines against infectious and non‐communicable diseases (Happi & Nkengasong, 2022). In particular, manufacturing capabilities for diagnostics could help Africa take control of its health as the diagnosis is important for determining therapy. Therefore, increasing the availability of reliable diagnostics is not only helpful in an effective therapeutic approach but also helps to increase the confidence among the population about medical facilities and health infrastructure. Rwanda and South Africa plan to incorporate the COVID‐19 vaccine into their standard immunization program; South Africa also intends to make COVID‐19 testing as routine as malaria and HIV testing.

The antiviral drug Paxlovid must be given within 5 days of the onset of COVID‐19 symptoms, which is nearly impossible in LMICs, where few tests and almost no rapid tests are available (Ledford & Maxmen, 2022). The available rapid tests could enable patients to test and treat themselves at home, reducing the demand on already overburdened health facilities. Despite that, African researchers have been refused access to Paxlovid for clinical trials in African populations based on Pfizer's agreement with the Medicines Patent Pool that compels companies to get Pfizer's approval before combining their generic Paxlovid with other treatments or supplying Paxlovid to researchers who want to examine combinations. This could put a stop to efforts to test the treatment in African populations and in combination with therapies that might potentially broaden its utility (Ledford & Maxmen, 2022).

Governments also need to invest in early‐warning systems for disease outbreaks, which could potentially protect millions of Africans as well as people on other continents. Moreover, thousands of African scientists, doctors, nurses, and other trained health workers have gone abroad for training or employment, and financial and occupational motivations are needed to entice them back. Training people in genomic sequencing, bioinformatics, and genomics will be crucial, particularly for recognizing, tracking, and understanding outbreaks of zoonotic origin such as COVID‐19 (Table 3). The Africa CDC has mandated the African Center of Excellence for Genomics of Infectious Diseases (ACEGID) in Nigeria with the task of sequencing all COVID‐19 samples from African Union member states that do not have sequencing capabilities, and it has already sequenced COVID‐19 samples from around 30 African countries. ACEGID has trained more than 1,300 geneticists and public health workers and officials from other countries in diagnostics and genomics for infectious diseases and more than 100 scientists from over 30 African countries on next‐generation sequencing of SARS‐CoV‐2. South Africa plans to expand its pathogen monitoring network using its pioneering genome sequencing network.

**Table 3:** Variants identified in Africa.

Date	SARS‐CoV‐2 VOCs	Number of mutations	Country
October 2020	Beta variant B.1.351	Total number of mutations: 21 Mutations in the Spike protein: 9	South Africa (Network for Genomic Surveillance) (NGS‐SA).
November 2021	Omicron variant B.1.1.529	Total number of mutations: app 50 Mutations in the Spike protein: 30	South Africa (Lancet Laboratories in Pretoria)

Lastly, vaccine production necessitates highly qualified staff with expertise in a wide range of specialties, some of which are specific to a given vaccine. The know‐how is often learned in vaccine manufacturing facilities, and most African countries, therefore, lack adequate training opportunities. Today, only between 2,000 and 3,000 sufficiently trained personnel work as full‐time employees, most of whom are for R&D organizations that are not solely focused on vaccines (African Union & Africa CDC, 2022). There are far too few degree programs related to vaccine manufacture, and those that do exist mostly focus on clinical training. Consequently, even graduates with relevant degrees are not sufficiently equipped with the necessary knowledge and technical abilities. It is predicted that US$10 billion are required over the next 10 years to fund capacity building for manufacturing plants, R&D institution, and regulatory agencies, as well as pre‐clinical and clinical trials for priority diseases (African Union & Africa CDC, 2022). This is why the mRNA vaccine technology transfer hub is so important as partners will provide training and financial assistance to develop the human capital required for production, quality control, and product regulation.

## Conclusion

African leaders must not miss this great opportunity to build the capacity for manufacturing vaccines, diagnostics, and therapeutics, an opportunity that could transform the African continent. This strategic investment could help Africa build a platform for producing vaccines against malaria, TB, HIV, and even cancer. Technology transfer, the profitability of the owner companies, and intellectual property must be discussed and resolved quickly, and organizations, such as COVAX, UNICEF, or the Global Alliance for Vaccines and Immunization (GAVI), should commit to purchasing vaccinations from local manufacturers to sustain the nascent industry on the continent. This, in turn, will bolster Africa's commitment to establishing its sovereignty over health and medicines. Additionally, governments and the media must create better awareness and understanding of the benefits of vaccines among the population. The reduction in vaccine hesitancy and more confidence in medical professionals will increase demand for vaccines, especially COVID‐19 vaccines, which in turn increases the production of vaccines. Ultimately, the ongoing endeavors to build and strengthen regional monitoring, regulatory and laboratory networks of the existing powerhouses in the continent are critical as they can help to raise overall standards and assist national reference centers and public health institutes.

## Author contributions

**AbdulRahman A Saied:** Conceptualization; Data Curation; Investigation; Visualization; Writing—Original Draft; Writing—review & editing. **Asmaa A Metwally:** Writing—Original Draft; Writing—review & editing. **Manish Dhawan:** Writing—Original Draft; Writing—review & editing. **Om Prakash Choudhary:** Writing—Original Draft; Writing—review & editing. **Hani Aiash:** Supervision; Visualization; Writing—Original Draft; Writing—review & editing.

## Disclosure statement and competing interests

The authors declare that they have no conflict of interest.
